# A new approach to *in silico *SNP detection and some new SNPs in the *Bacillus anthracis *genome

**DOI:** 10.1186/1756-0500-4-114

**Published:** 2011-04-08

**Authors:** Andrzej K Brodzik, Joe Francoeur

**Affiliations:** 1 The MITRE Corporation, 202 Burlington Road, Bedford, MA 01730, USA

**Keywords:** *Bacillus anthracis*, cyclic difference sets, DNA sequence homology assessment, DNA sequence markers, SNP, strain comparison

## Abstract

**Background:**

*Bacillus anthracis *is one of the most monomorphic pathogens known. Identification of polymorphisms in its genome is essential for taxonomic classification, for determination of recent evolutionary changes, and for evaluation of pathogenic potency.

**Findings:**

In this work three strains of the *Bacillus anthracis *genome are compared and previously unpublished single nucleotide polymorphisms (SNPs) are revealed. Moreover, it is shown that, despite the highly monomorphic nature of *Bacillus anthracis*, the SNPs are (1) abundant in the genome and (2) distributed relatively uniformly across the sequence.

**Conclusions:**

The findings support the proposition that SNPs, together with indels and variable number tandem repeats (VNTRs), can be used effectively not only for the differentiation of perfect strain data, but also for the comparison of moderately incomplete, noisy and, in some cases, unknown *Bacillus anthracis *strains. In the case when the data is of still lower quality, a new DNA sequence fingerprinting approach based on recently introduced markers, based on combinatorial-analytic concepts and called cyclic difference sets, can be used.

## 

*I have deeply regretted that I did not proceed far enough at least to understand something of the great leading principles of mathematics; for men thus endowed seem to have an extra sense*.

Charles Darwin

## Background

This research is part of an effort to develop novel techniques for the interrogation of pathogenic genomes. In this domain the task of *Bacillus anthracis *strain differentiation poses a particularly difficult challenge [1-4]. Since most *B. anthracis *strains are highly monomorphic, sequence typing must rely on subtle differences between genomes, sampled at multiple loci [5]. The complexity of the problem will increase in cases where only partial sequence data is available, or sequences contain errors, and as design of engineered bacterial genomes becomes possible [6].

The principal genomic markers used in sequence typing are VNTRs, indels and SNPs. The occurrence of VNTRs and indels in the *B. anthracis *genome in the three strains considered here was recently investigated in [7]. Here, we undertake the analysis of SNPs. The use of SNPs in both human and microbial DNA investigations has a long tradition [8]. The advantages of SNPs include high concentration in coding regions, fixed length, and lower susceptibility to short read sequencing errors than VNTRs. In applications these advantages must be balanced against SNPs' relatively slow mutation rates and relatively low resolving power. In cases when sequence typing by SNPs is not sufficient, the use of SNPs in combination with other markers should be considered [9].

In this work the occurrence of SNPs is investigated in the three main strains of the *B. anthracis *genome: Ames Ancestor, Ames and Sterne. It is shown that SNPs are abundant in the *B. anthracis *genome and that they are distributed relatively uniformly throughout the sequence. These findings demonstrate that the *B. anthracis *SNPs can be used effectively as part of an increased resolution, multi-tier strain differentiation scheme for the analysis of moderately incomplete, noisy or uncertain data. The SNP detection approach used here is based on an advanced design theory construction known as the cyclic difference set [10]. In this approach the comparison of DNA sequences is replaced by the comparison of cyclic difference set distributions associated with these sequences. The similarity of these distributions is used first to assess DNA sequence homology and subsequently to identify indels and SNPs. The cyclic difference set approach has many advantages [7]; the primary one, which is particularly relevant to this work, is that it permits a high degree of flexibility in selecting an appropriate sequence variation resolution that can be adapted to a given application.

The work described here intersects several application domains. Prior work on *B. anthracis *includes [7,1,5,11,3], and [12-14]. Prior work on bacterial genome structure includes [15-18]. Prior work on SNP taxonomy and detection includes [8,19,1], and [20]. Prior work on cyclic difference sets includes [10] and [21-23].

### Data

The *B. anthracis *genome is made up of chromosomal DNA and two plasmids, pXO1 and pXO2. We analyzed the chromosomal sequences of Ames Ancestor GenBank: NC_007530.2, Ames GenBank: NC_003997.3, and Sterne GenBank: NC_005945.1, the pXO1 plasmid sequences of Ames Ancestor GenBank: NC_003980 and Sterne GenBank: NC_001496, and the pXO2 plasmid sequences of Ames Ancestor GenBank: NC_003981.1 and Pasteur GenBank: NC_012659.1. For brevity, we refer to Ames Ancestor, Ames, Sterne, and Pasteur as AA, A, S, and P.

### SNP definition and taxonomy

There is no standard, mathematically consistent definition of the term SNP [8]. We consider it *essential *to establish such a definition, so that confusion can be avoided in analysis, in comparison of results and in discussions. In this work a SNP is defined as a single letter difference between two sequences flanked on the left and on the right by at least one letter that is identical in both sequences. For example, in the strings

A **C **G **T **A **CG **T

A **A **G **G **A **TT **T

the second and fourth letters are SNPs but the sixth and seventh letters are indels, as the letter differences are adjacent. This convention is different from general practice, which sometimes permits adjacent letter differences to be regarded as SNPs [8]. We insert the non-adjacency constraint into the SNP definition because: (1) such modification permits mathematically unambiguous separation of SNPs and indels, and (2) such separation is biologically meaningful as adjacent and closely spaced SNPs often coincide with large indels.

The definition of SNP must be further disambiguated when more than two sequences are considered. In this case two or more distinct letters might appear at a putative SNP position, raising the possibility of counting each pair-wise mismatch as a separate SNP. We will ignore this multiplicity. For example, both triples A-C-T and A-C-C will be considered instances of a single SNP. We will distinguish between coding and non-coding SNPs, and between synonymous and non-synonymous SNPs (the latter referred to as *nsSNPs*). In a three-way comparison a coding SNP is considered non-synonymous when at least one of the pair-wise SNPs is non-synonymous. For example, there are two pair-wise SNPs in letters A-C-C in the three-way comparison of AA-A-S, one for the pair of strains AA-A and one for the pair of strains AA-S. If either of these pair-wise SNPs is non-synonymous then the three-way SNP is declared an nsSNP.

### Approach

The analysis of the *B. anthracis *genome was performed using the approach described in [7]. Here, we will give only a brief overview of this approach as it is relevant to SNPs. The algorithm consists of two main stages: indel detection and SNP detection. In the first stage the occurrences of certain short quasi-random strings, called cyclic difference sets (DSs), in two homologous DNA sequences are identified and, subsequently, the locations of these occurrences are compared. The algorithm proceeds as follows:

• In each of the two DNA sequences being compared identify the consecutive occurrences of a selected DS. For example, choosing the DS, 1101000, the DNA sequences

ACCGCTTACACCACGGGGCCACAGTCCTCTTT...

ACCGCATACACCACGGCCACAGTCCTCTTTAG...

give rise to the DS sequences associated with the nucleotide C,

01000000001000000010000001000000...

01000000001000001000000100000000...

• Convert the above DS sequences to shorter sequences of inter-DS gaps,

876...

856...

• Align the gap sequences and identify the mismatching strings of gaps, 7 and 5, or (CAC)GGGG and (CAC)GG.

The rationale for using DSs as sequence markers is that when DNA sequences are highly homologous, so are the sequences of DS locations. Conversely, in regions where DNA sequences differ, so do the DS sequences. This is convenient as the analysis of DNA sequences can then be replaced by the analysis of much sparser, and therefore easier to compute, DS sequences. Since a difference in DS sequences marks the occurrence of an indel, mismatching segments are removed from the DS sequences.

In the second stage of the algorithm, the DS sequences are mapped back to "new", indel-free DNA sequences. These DNA sequences differ only by nucleotide mismatches. Once adjacent mismatches are filtered, SNPs are easily identified by a point-wise comparison of the modified nucleotide sequences. In the example given above this yields the indel-free sequences

ACCGC**T**TACACCACCCACAGTCCTCTTT...

ACCGC**A**TACACCACCCACAGTCCTCTTT...

Point-wise comparison of these sequences reveals a SNP T/A at the 6^th ^bp.

Several comments are necessary here to make statements precise. First, while a more natural acronym for a cyclic difference set would be CDS, to avoid potential confusion with a coding sequence we settle for DS. Second, DSs are combinatorial designs that are *associated *with, not identical to, the special binary strings considered here. However, for convenience and by abuse of language in this text we will refer to the relevant strings as DSs. While motivating the technical approach, for brevity, we mention here only the computational complexity reason for the utility of DSs.

Specifically, the computational advantage of the method as compared to a direct approach not relying on DSs is proportional to the abundance of DSs in genomes (1 in 500 nucleotides in the B. anthracis genome). This advantage is further enhanced by the suitability of the method for implementation using Fast Fourier Transform algorithm, which requires only *n log_2 _n *complex operations. For a more extensive discussion of the role of DSs in DNA sequence analysis the reader is directed to [7].

## Results

**Figure 1:**
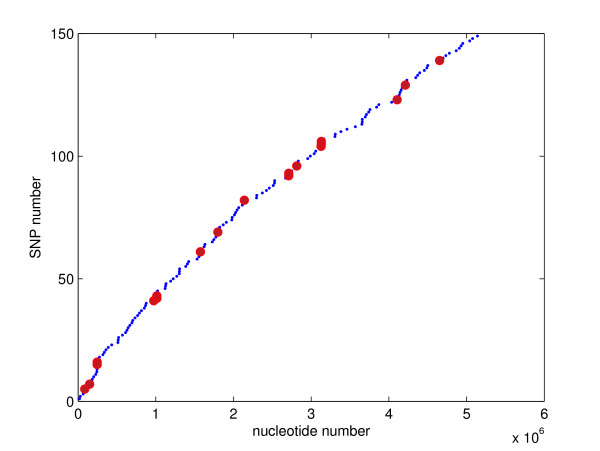
**Distribution of SNPs in chromosomal sequences of the *B. anthracis *genome (A-S)**. Small blue dots mark AA-S SNPs, large red dots mark AA-A SNPs.

**Figure 2:**
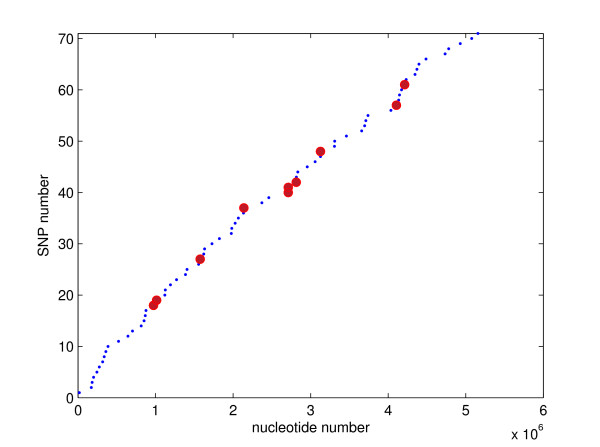
**Distribution of nsSNPs in chromosomal sequences of the *B. anthracis *genome (A-S)**. Small blue dots mark AA-S SNPs, large red dots mark AA-A SNPs.

**Figure 3:**
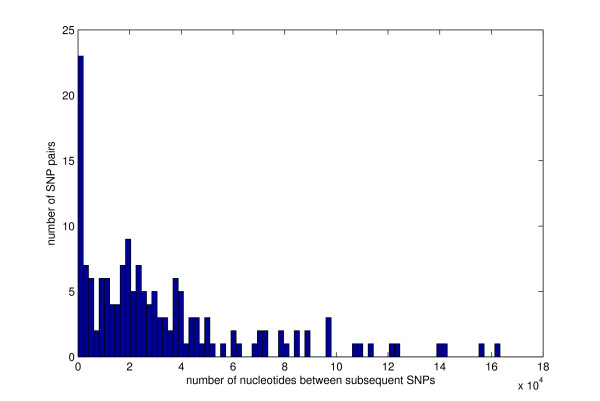
**Histogram of distances between subsequent SNPs in the *B. anthracis *chromosome**. The minimum, average and maximum distance between subsequent SNPs is 2, 34499 and 163349 bp, respectively, however many SNPs are less than 2000 bp apart.

The results of the SNP analysis of the *B. anthracis *genome are summarized in Tables 1 and 2. The distributions of the chromosomal SNPs (all and non-synonymous) are shown in Figures 1 and 2. The histogram of distances between subsequent chromosomal SNPs is shown in Figure 3. A list of all SNPs annotated for position, nucleotide letter, coincidence with a coding region, and protein preservation is included in [Additional file 1].

**Table 1:** Abundance and taxonomy of SNPs in Ames Ancestor, Ames and Sterne genomes reported in [13] and computed using the DS approach.

sequence	Read	DS	coding	ns
**Chromosome (AA-S)**	-	131	90	62
**Chromosome (AA-A)**	2	19	11	10
**Chromosome (A-S)**	-	150	101	78
**Chromosome (AA-A-S)**	-	150	101	78
**pXO1 (AA-S)**	15*	14	7	6
**pXO2 (AA-P)**	21	21	16	9

Hyphens denote that results for a relevant strain comparison were not published. Asterisk denotes that adjacent SNPs, not considered here, were reported (see the discussion of SNPs in Section 3).

**Table 2:** Distribution of SNPs in Ames Ancestor, Ames, and Sterne genomes.

sequence	strain homology	SNP spacing (average)	SNP spacing (adjusted for indels)
**Chromosome (AA-S)**	99.96%	40.3	40.3
**Chromosome (AA-A)**	100.00%	277.8	277.8
**Chromosome (A-S)**	99.94%	34.5	34.5
**pXO1 (AA-S)**	72.38%	13.0	9.4
**pXO2 (AA-P)**	98.49%	4.5	4.4

The average SNP spacing, given in Kbp, is computed by dividing the sequence length by the number of SNPs. Non-indel SNP spacing is computed similarly, except that the lengths of all indels and polymorphic regions (SNP clusters, i.e. regions where average SNP spacing is greater than one in every twenty bases) are subtracted from the total sequence length.

The chromosomal analysis included the three pair-wise comparisons of AA-S, AA-A and A-S. These comparisons revealed 131, 19 and 150 SNPs, respectively (Table 1). The SNPs found in the AA-S and AA-A strain comparisons partition the SNPs found in the A-S strain comparison. This suggests that Ames and Sterne are both descendants of Ames Ancestor. The relatively large number of SNPs in AA-S confirms that AA is evolutionarily more distant from S than from A [1]. About 70% of chromosomal SNPs are coding and about 80% of coding SNPs are non-synonymous. The ratio of all coding SNPs to all SNPs is 67%. This ratio is only modestly lower than the ratio of coding DNA and the entire genome sequence lengths, 78% in the AA strain. This result suggests that there is a similar degree of sequence conservation in the two sequence types. Both SNPs and nsSNPs are relatively uniformly distributed along the chromosome (Figures 1 and 2). The minimum, average and maximum distance between subsequent A-S SNPs is 2, 34499 and 163349 bp, respectively, although many SNPs are less than 2000 bp apart (Figure 3, Table 2). Interestingly, despite the close proximity of several pairs of SNPs, only the SNPs 93 and 94 occur within the same gene. The distributions of SNPs are only negligibly affected by the occurrence of indels. This is so because chromosomal sequences are highly homologous: the AA-A comparison yields only two multi-base indels, a 123-base-long indel at 1151242 bp and a 10-base-long indel at 2612043 bp; the AA-S comparison yields a single 100-base long indel at 4147353 bp (all locations are given in the AA coordinates) [7].

The plasmid analysis included pair-wise comparisons of strains AA-S for pXO1 and AA-P for pXO2. Given their relatively short sequence lengths, the pXO1 and pXO2 plasmids are polymorphism-rich, containing 14 and 21 SNPs each, respectively. Of these SNPs, 7 and 16 are coding SNPs. Of the coding SNPs 6 and 9 are nsSNPs. The minimum, average and maximum distance between subsequent SNPs in the pXO1 plasmid are 3, 12977 and 84568 bp. The minimum, average and maximum distance between subsequent SNPs in the pXO2 plasmid are 94, 4516 and 13884 bp. The density of SNPs decreases in the pXO1 and pXO2 plasmids when indels are removed from the sequences (Table 2). The effect is most pronounced in the pXO1 sequence, due to the occurrence of two large indels at 43348-48589 and 117228-162050 bp.

Overall, when adjusted for indels, SNPs are distributed, rather surprisingly, in a relatively uniform fashion across the entire *B. anthracis *genome, but with varying inter-SNP spacing in each of the three sequences.

## Conclusions

This work describes the structure of *B. anthracis *SNPs arising from *in silico *comparison of the Ames Ancestor, Ames and Sterne strains. This result complements the characterization of *B. anthracis *indels given in [7] and extends the analysis given in [13] in both the number of SNPs identified and the information provided about their type and distribution. While a later work, [24], slightly extends the results of [13], it does so only with respect to the 12 so-called canonical SNPs.

Indels and SNPs, together with VNTRs (The distinction between indels and VNTRs is made for historical reasons; mathematically, VNTR is a special case of indel), capture all sequence differences in pan-genomes (Pan-genome is a superset of all the genes in all the strains of a species [16]. More generally, pan-genome can be defined as a reference genome for a species plus the superset of all the genomic variants occurring in all the strains.). Knowledge of these differences can be used either to address basic biological research problems, e.g., investigation of genomic function and evolutionary processes [12], or in applications such as strain fingerprinting [1] and monitoring of DNA sequence synthesis orders [25]. In each of these problems selecting the appropriate granularity of analysis is one of the main decisions that must be made in experiment design.

While it was previously suggested that many *B. anthracis *strains, including the ones considered here, can be identified using certain minimal sets of markers, such as the so-called *canonical *SNPs [5] or special sets of VNTRs [2], such approaches are certain to be effective only when the strain is known and the data is perfect. This might not always be the case. Indeed, in many practical sequence analysis scenarios the data can be Large (whole genome), Uncertain (a new strain), Noisy (contaminated at the source, corrupted in the process of data collection, sequencing or sequence assembly, or purposefully engineered), or Incomplete (LUNI). In these cases a minimum set of markers will not, in general, suffice to identify all strains, and higher resolution approaches, relying on sequence over-sampling, must be employed.

Results of the SNP investigation undertaken here together with the prior work on DSs [7] both inform the design and suggest a certain organization of these approaches (Table 3). As mentioned before, the most parsimonious and - at the same time - the most error-prone strategy for strain differentiating is based on a minimal set of SNPs. This set needs to contain at least n SNPs to be able to differentiate 2^n ^strains, provided the data is of sufficient quality to accurately represent the required SNPs. One can improve the resolution of this scheme, at the cost of increasing its complexity, by extending the minimal set of SNPs to the set of all known standard genomic differences. Aided by a roughly ten to hundred-fold increase (depending on the strains under consideration) in the sampling rate, this approach can be expected to be effective in the case of closely related strains whose sequence data is of moderate quality or partly unavailable (which might include sequence segments containing SNPs from the minimal set). Exceptionally complex tasks, such as detection of data manipulation or revelation of unknown distant strains, will require the use of even more dense, uniform and flexible sequence sampling schemes. One such scheme is offered by the DS-based sequence homology assessment procedure [7]. In this approach the average marker spacing can be selected from the range of tens to tens of thousands of nucleotides. This approach will be effective in all but the most challenging sequence analysis scenarios.

**Table 3:** DNA sequence fingerprinting scheme choices for three strains of the *B. anthracis *chromosomal sequence ordered in terms of increasing sequence resolution.

marker	# of markers	detectable strains	data quality
**Minimal set of SNPs**	2	known	perfect
**All SNPs + VNTRs**	150+15	some unknown	moderate
**CDSs**	~10,000	many unknown	poor
**Sequence alignment**	~5,300,000	arbitrary	arbitrary

## Competing interests

The authors declare that they have no competing interests.

## Authors' contributions

AKB conceived the approach. AKB and JF implemented and tested the method and wrote the manuscript. Both authors read and approved the final manuscript.

## Supplementary Material

Additional file 1 **Tables of SNPs**. Tables of SNPs for chromosomal and plasmid sequences of *B. anthracis *strains Ames Ancestor, Ames, Sterne, and Pasteur. The GenBank reference numbers of sequences are given in the Data section. Click here for file

## References

[B1] Keim P Van Ert MN Pearson T Vogler AJ Huynh LY Wagner DM Anthrax molecular epidemiology and forensics: using the appropriate marker for different evolutionary scales Infection genetics and evolution 2004 4 205 213 10.1016/j.meegid.2004.02.005 15450200

[B2] Lista F Faggioni G Valjevac A Ciammaruconi A Vaissaire J le Doujet C Gorgé O De Santis R Carattoli A Ciervo A Fasanella A Orsini F D'Amelio R Pourcel C Cassone A Vergnaud G Genotyping of bacillus anthracis strains based on automated capillary 25-loci multiple locus variable number tandem repeats analysis BMC Microbiology 2006 6 1 15 10.1186/1471-2180-6-33 16600037 PMC1479350

[B3] Marston CK Gee JE Popovic T Hoffmaster AR Molecular approaches to identify and differentiate *Bacillus anthracis *from phenotypically similar bacillus species isolates BMC Microbiology 2006 6 22 28 10.1186/1471-2180-6-22 16515693 PMC1413540

[B4] Pallen MJ Nelson KE Preston GM Bacterial pathogenomics 2007 Washington DC: ASM Press

[B5] Keim P Pearson T Okinaka R Microbial forensics: DNA fingerprinting of *Bacillus anthracis* Anal Chem 2008 4 4791 4799 10.1021/ac086131g 18609763

[B6] Gibson DG Glass JL Lartigue C Noskov VN Chuang RY Algire MA Benders GA Montague MG Ma L Moodie MM Merryman C Vashee S Krishnakumar R Assad-Garcia N Andrews-Pfannkoch C Denisova EA Young L Qi ZQ Segall-Shapiro TH Calvey CH Parmar PP Hutchison CA III Smith HO Venter JC Creation of a bacterial cell controlled by a chemically synthesized genome Science 2010 329 52 56 10.1126/science.1190719 20488990

[B7] Brodzik AK Rapid Sequence Homology Assessment by Subsampling the Genome Space Using Difference Sets IEEE Transactions on Information Theory, Special Issue on Molecular Biology and Neuroscience 2010 56 2 756 770

[B8] Brookes AJ The essence of SNPs Gene 1999 234 177 186 10.1016/S0378-1119(99)00219-X 10395891

[B9] Brodzik AK Quaternionic periodicity transform: an algebraic solution to the tandem repeat detection problem Bioinformatics 2007 23 694 700 10.1093/bioinformatics/btl674 17237057

[B10] Baumert LD Cyclic difference sets 1971 Berlin: Springer

[B11] Keim P Grundike JM Klevytska AM Schupp JM Challacombe J Okinaka R The genome and variation of *Bacillus anthracis* Molecular Aspects of Medicine 2009 30 397 405 10.1016/j.mam.2009.08.005 19729033 PMC3034159

[B12] Pilo P Perreton V Frey J Molecular epidemiology of *Bacillus anthracis*: determining the correct origin Appl and Environ Mirobiol 2008 74 2928 31 10.1128/AEM.02574-07 PMC2394883 18326672

[B13] Read TD Salzberg SL Pop M Shumway M Umayam L Jiang L Holtzapple E Busch JD Smith KL Schupp JM Solomon D Keim P Fraser CM Comparative genome sequencing for discovery of novel polymorphisms in *Bacillus anthracis* Science 2002 296 2028 2033 10.1126/science.1071837 12004073

[B14] Kolsto A-B Tourasse NJ Okstad OA What sets *Bacillus anthracis *apart from other Bacillus species? Annual Rev Microbiol 2009 63 451 476 10.1146/annurev.micro.091208.073255 19514852

[B15] Cummings CA Relman DA Microbial forensics - cross-examining pathogens Science 2002 296 1976 1979 10.1126/science.1073125 12004075

[B16] Konstantinidis KT Ramette A Tiedje JM The bacterial species definition in the genomic era Philosophical Transactions of The Royal Society B 2006 361 1929 40 10.1098/rstb.2006.1920 PMC1764935 17062412

[B17] Frazer C Alm EJ Polz MF Spratt BG Hanage WP The bacterial species challenge: making sense of genetic and ecological diversity Science 2009 323 741 6 10.1126/science.1159388 19197054

[B18] Freeman JM Plasterer TN Smith TF Mohr SC Patterns of genome organization in bacteria Science 1998 279 1827a 10.1126/science.279.5358.1827a

[B19] Mooney S Bioinformatics approaches and resources for single nucleotide polymorphism functional analysis Briefings in Bioinformatics 2005 6 44 56 10.1093/bib/6.1.44 15826356

[B20] Xu Y Gogarten JP Computational methods for understanding bacterial and archeal genomes 2008 Singapore: Imperial College Press

[B21] Colbourn CJ Dinitz JH Handbook of combinatorial designs 2006 New York: Chapman and Hall/CRC

[B22] Erdos P Turan P On a problem of Sidon in additive number theory J London Math Soc 1941 3 212 215 10.1112/jlms/s1-16.4.212

[B23] Sidon S Ein Satz uber trigonometrische Polynome und seine Anwendung in der Theorie der Fourier-Reihen Math Ann 1932 106 536 539 10.1007/BF01455900

[B24] Van Ert MN Easterday WR Huynh LY Okinaka RT Hugh-Jones ME Ravel J Global genetic population structure of Bacillus anthracis PLoS ONE 2007 5 1 10 10.1371/journal.pone.0000461 PMC1866244 17520020

[B25] Carlson R The changing economics of DNA synthesis Nature Biotechnology 2009 27 1091 4 10.1038/nbt1209-1091 20010582

