# Supplementary material for: A new approach to in silico SNP detection and some new SNPs in the Bacillus anthracis genome
Source: BMC Res Notes. 2011 Apr 8;4:114. doi: 10.1186/1756-0500-4-114 (PMC3094368; doi:10.1186/1756-0500-4-114)
Supplement: Additional file 1 — Tables of SNPs. Tables of SNPs for chromosomal and plasmid sequences of B. anthracis strains Ames Ancestor, Ames, Sterne, and Pasteur. The GenBank reference numbers of sequences are given in the Data section. [file 1756-0500-4-114-S1.PDF]

Supplemental Materials: Tables of SNPs

Andrzej K. Brodzik and Joe Francoeur

Table 1: SNPs for Ames Ancestor (AA), Ames (A), and Sterne (S) Chromosome Sequences. In the nsSNP column Y denotes nsSNP, N denotes sSNP and X denotes a non-coding SNP.

	position			value			coding	nsSNP	
index	AA	A	S	AA	A	S	AA	AA-A	AA-S
1	17294	17294	17295	t	t	c	Y	-	Y
2	28975	28975	28976	g	g	a	-	-	X
3	64632	64632	64633	t	t	c	-	-	X
4	73738	73738	73739	t	t	c	Y	-	N
5	84725	84725	84726	t	c	t	-	X	-
6	122896	122896	122897	g	g	a	-	-	X
7	148380	148380	148381	t	c	t	-	X	-
8	171823	171823	171824	c	c	t	Y	-	Y
9	182106	182106	182107	c	c	t	Y	-	Y
10	200731	200731	200732	c	c	t	Y	-	Y
11	226553	226553	226566	c	c	g	-	-	X
12	240050	240050	240063	t	t	c	Y	-	N
13	243867	243867	243880	c	c	t	Y	-	Y
14	245190	245190	245203	c	c	t	-	-	X
15	245489	245489	245502	t	g	t	-	X	-
16	245498	245498	245511	t	g	t	-	X	-
17	268670	268670	268683	c	c	t	-	-	X
18	274236	274236	274249	c	c	a	Y	-	Y
19	318187	318187	318200	c	c	t	Y	-	Y
20	337241	337241	337254	t	t	c	Y	-	Y
21	359536	359536	359549	g	g	a	Y	-	Y
22	387637	387637	387650	g	g	a	Y	-	Y
23	433277	433277	433320	g	g	t	-	-	X
24	513052	513052	513095	c	c	t	-	-	X
25	515111	515111	515154	g	g	a	Y	-	N
26	521963	521963	522006	a	a	g	Y	-	Y
27	571199	571199	571242	g	g	a	-	-	X
28	610254	610254	610297	g	g	a	-	-	X
29	629696	629696	629739	c	c	t	Y	-	N
30	644625	644625	644667	t	t	c	Y	-	Y

	position			value			coding	nsSNP	
index	AA	A	S	AA	A	S	AA	AA-A	AA-S
31	665308	665308	665380	t	t	c	Y	-	N
32	692932	692931	692827	c	c	t	Y	-	N
33	702761	702760	702656	g	g	c	Y	-	Y
34	732173	732172	732068	a	a	t	-	-	X
35	765499	765498	765394	a	a	c	Y	-	N
36	789298	789297	789193	g	g	a	Y	-	N
37	814684	814683	814579	t	t	g	Y	-	Y
38	852207	852206	852101	t	t	g	Y	-	Y
39	867050	867049	866944	a	a	c	Y	-	Y
40	877078	877077	876972	g	g	t	Y	-	Y
41	973706	973705	973600	c	a	c	Y	Y	-
42	1013521	1013520	1013415	c	t	c	Y	N	-
43	1013528	1013527	1013422	g	c	g	Y	Y	-
44	1017327	1017327	1017220	a	a	g	Y	-	N
45	1023150	1023150	1023043	a	a	g	-	-	X
46	1120096	1120096	1119988	c	c	t	Y	-	Y
47	1125558	1125558	1125450	a	a	g	Y	-	Y
48	1131906	1131906	1131798	t	t	c	-	-	X
49	1191432	1191310	1191378	t	t	c	Y	-	Y
50	1223198	1223076	1223144	c	c	t	-	-	X
51	1270555	1270432	1270501	g	g	t	Y	-	Y
52	1301762	1301639	1301708	t	t	c	-	-	X
53	1303210	1303087	1303156	g	g	a	Y	-	N
54	1306861	1306738	1306807	c	c	t	Y	-	N
55	1385721	1385598	1385667	g	g	a	Y	-	Y
56	1406212	1406089	1406158	c	c	t	Y	-	Y
57	1423420	1423297	1423366	a	a	g	Y	-	N
58	1531733	1531610	1531687	g	g	a	Y	-	N
59	1555385	1555262	1555337	a	a	g	Y	-	Y
60	1572965	1572842	1572917	t	t	c	Y	-	N
61	1574662	1574539	1574614	c	t	c	Y	Y	-
62	1592398	1592275	1592350	c	c	a	-	-	X
63	1621512	1621389	1621464	c	c	a	Y	-	Y
64	1631941	1631818	1631893	g	g	t	Y	-	Y
65	1728049	1727926	1727994	g	g	a	Y	-	Y
66	1747193	1747070	1747138	a	a	g	Y	-	N
67	1774177	1774054	1774121	c	c	t	-	-	X
68	1797708	1797585	1797652	t	t	c	-	-	X
69	1798709	1798586	1798653	a	g	a	-	X	-
70	1798769	1798646	1798713	g	g	t	-	-	X
71	1823153	1823030	1823097	g	g	c	Y	-	Y
72	1867996	1867873	1867940	t	t	c	Y	-	N
73	1907366	1907242	1907310	t	t	a	-	-	X
74	1977849	1977725	1977795	c	c	t	Y	-	Y
75	1982986	1982862	1982932	c	c	t	Y	-	Y
76	2012193	2012069	2012139	t	t	c	-	-	X
77	2026733	2026609	2026637	t	t	c	Y	-	Y
78	2045712	2045588	2045616	a	a	c	Y	-	N
79	2067935	2067811	2067839	g	g	a	Y	-	Y
80	2113910	2113786	2113814	c	c	t	Y	-	N

	position			value			coding	nsSNP	
index	AA	A	S	AA	A	S	AA	AA-A	AA-S
81	2133680	2133556	2133584	g	g	t	Y	-	Y
82	2139224	2139100	2139128	c	t	c	Y	Y	-
83	2295848	2295724	2295775	g	g	a	Y	-	N
84	2299647	2299523	2299574	a	a	g	-	-	X
85	2371243	2371119	2371172	t	t	c	Y	-	Y
86	2423657	2423533	2423585	a	a	g	-	-	X
87	2460413	2460289	2460341	g	g	t	Y	-	Y
88	2511427	2511303	2511356	a	a	g	-	-	X
89	2528887	2528763	2528817	t	t	a	-	-	X
90	2529804	2529680	2529734	a	a	g	-	-	X
91	2668966	2668838	2669302	c	c	t	Y	-	N
92	2711421	2711293	2711756	a	g	a	Y	Y	-
93	2711476	2711348	2711811	t	c	t	Y	Y	-
94	2749671	2749543	2750006	a	a	g	-	-	X
95	2805282	2805154	2805620	t	t	c	-	-	X
96	2814306	2814178	2814644	t	g	t	Y	Y	-
97	2815575	2815447	2815913	c	c	t	Y	-	Y
98	2834318	2834190	2834656	g	g	a	Y	-	Y
99	2956135	2956007	2956699	g	g	a	Y	-	Y
100	2989856	2989728	2990419	g	g	a	Y	-	N
101	3038888	3038760	3039451	t	t	c	-	-	X
102	3057853	3057725	3058419	g	g	a	Y	-	Y
103	3126496	3126368	3127062	c	c	t	Y	-	Y
104	3127419	3127291	3127985	a	c	a	Y	Y	-
105	3130006	3129878	3130572	a	g	a	-	X	-
106	3130008	3129880	3130574	t	a	t	-	X	-
107	3163858	3163731	3164424	a	a	g	Y	-	N
108	3306423	3306296	3306990	c	c	t	Y	-	Y
109	3310574	3310447	3311141	g	g	a	Y	-	Y
110	3382079	3381952	3382646	c	c	t	Y	-	N
111	3460779	3460652	3461354	t	t	c	Y	-	Y
112	3568849	3568722	3569423	a	a	g	-	-	X
113	3653651	3653524	3654219	a	a	g	Y	-	N
114	3657369	3657242	3657937	a	a	g	Y	-	N
115	3657516	3657389	3658084	g	g	a	Y	-	Y
116	3696168	3696041	3696736	c	c	t	Y	-	Y
117	3712048	3711921	3712616	t	t	c	Y	-	Y
118	3739850	3739723	3740418	a	a	g	Y	-	Y
119	3758015	3757888	3758388	a	a	g	Y	-	N
120	3841816	3841689	3842189	g	g	a	-	-	X

	position			value			coding	nsSNP	
index	AA	A	S	AA	A	S	AA	AA-A	AA-S
121	3871854	3871727	3872227	c	c	g	-	-	X
122	4035203	4035076	4035575	t	t	c	Y	-	Y
123	4106512	4106385	4106886	g	t	g	Y	Y	-
124	4126060	4125933	4126434	t	t	c	Y	-	N
125	4134667	4134540	4135041	t	t	c	Y	-	Y
126	4145299	4145172	4145673	c	c	t	Y	-	Y
127	4158968	4158841	4159343	c	c	t	Y	-	N
128	4172169	4172042	4172544	g	g	t	Y	-	Y
129	4212867	4212740	4213239	g	a	g	Y	Y	-
130	4221445	4221318	4221817	g	g	a	Y	-	N
131	4232753	4232626	4233125	c	c	t	Y	-	Y
132	4345557	4345430	4346119	t	t	a	Y	-	Y
133	4371291	4371164	4371853	a	a	g	Y	-	Y
134	4396547	4396420	4397109	g	g	c	Y	-	Y
135	4456896	4456769	4457458	g	g	a	-	-	X
136	4488395	4488268	4488957	g	g	a	Y	-	Y
137	4501001	4500874	4501563	g	g	a	-	-	X
138	4624259	4624132	4625355	t	t	c	-	-	X
139	4653513	4653387	4654609	t	g	t	-	X	-
140	4698084	4697958	4699293	t	t	c	-	-	X
141	4733700	4733574	4734909	a	a	g	Y	-	Y
142	4779524	4779398	4780733	t	t	c	Y	-	Y
143	4867375	4867249	4868586	g	g	a	-	-	X
144	4906434	4906308	4907645	a	a	g	-	-	X
145	4929312	4929186	4930523	t	t	g	Y	-	Y
146	4951610	4951484	4952821	g	g	a	-	-	X
147	5040523	5040397	5041732	c	c	t	-	-	X
148	5077771	5077645	5078980	g	g	a	Y	-	Y
149	5140491	5140365	5141736	g	g	a	-	-	X
150	5157810	5157684	5159055	g	g	a	Y	-	Y

Table 2: SNPs for Ames Ancestor (AA) and Sterne (S) pX01 Sequences.

	position		value		coding	nsSNP
index	AA	S	AA	S		
1	7452	7318	c	t	Y	-
2	16661	16527	c	g	Y	Y
3	36550	36499	t	g	-	-
4	36553	36502	a	t	-	-
5	42162	42111	c	t	-	-
6	42198	42147	a	t	-	-
7	63143	63092	c	g	Y	Y
8	69013	68962	g	c	-	-
9	69031	68980	g	c	Y	Y
10	74072	74021	g	a	Y	Y
11	77518	77467	a	g	Y	Y
12	162086	162042	g	a	-	-
13	179699	179676	t	c	-	-
14	180878	180855	g	a	Y	Y

Table 3: SNPs for Ames Ancestor (AA) and Pasteur (P) pX02 Sequences.

	position		value		coding	nsSNP
index	AA	P	AA	P		
1	8639	9133	c	t	Y	-
2	9106	9600	t	g	Y	Y
3	14036	14529	t	c	Y	-
4	16828	17321	g	c	Y	Y
5	19918	20410	a	g	Y	-
6	31027	31517	g	a	Y	Y
7	37496	37994	t	c	Y	Y
8	37950	38448	c	t	Y	-
9	38347	38845	g	a	Y	Y
10	42025	42523	c	t	Y	Y
11	42119	42617	a	t	Y	Y
12	42759	43257	g	a	-	-
13	51777	52267	a	t	Y	-
14	52693	53183	c	t	Y	Y
15	57326	57816	a	g	Y	-
16	61771	62243	t	c	Y	Y
17	63239	63711	c	a	-	-
18	66865	67335	c	a	-	-
19	72925	73395	t	g	-	-
20	76409	76883	a	t	-	-
21	80793	81267	g	a	Y	-
